# Supplementary material for: Videogame exposure positively associates with selective attention in a cross-sectional sample of young children
Source: PLoS One. 2021 Sep 27;16(9):e0257877. doi: 10.1371/journal.pone.0257877 (PMC8476027; doi:10.1371/journal.pone.0257877)
Supplement: S1 Table — (DOCX) [file pone.0257877.s001.docx]

| **S1 Table.** List of videogames played by participants. | | |
| --- | --- | --- |
| **Fast-Reaction Games** | **Slow-Reaction Games** | |
| AG Drive | ABC Français | Magic Kinder App |
| Amazing World | ABC Game | Math Dream Box |
| Aquarium | Amazing Match | Mathletics |
| Block City Wars | Amazing Shape Puzzle | Mermaid Glam |
| Bugs and Buttons | Angry Birds | Mickey’s Illusions |
| Bus Driver | Animal Phone | Minecraft |
| Castle Crashers | Baby Doctor | Mini Bows Boutique |
| City Driving | Baby Dress Up | Monkey Math |
| Crabby | Barbie Life | Monster Math |
| Crossy Roads | Betty Crocker Baking | Monument Valley |
| Cut the Rope | Bible for Kids | Movie Star Planet |
| Dino Train | BlockCraft | Ms. Hollywood |
| Disney Infinity | Blooming Blossom | My Little Pony |
| Doodle Jump | Bubble Guppies | My Little Pony Quest |
| Dumb Ways to Die | Candy Crush | My Little Pony: Friendship Gardens |
| Exploration | Candy Paradise | My Talking Angela |
| Feed Me! | Cinderella FreeFall | My Talking Tom |
| Fish Predator | Clash of the Clans | New Girl |
| Frogger | Coloring 123 | Osmo Number |
| Geometry Dash | Cool Math Games | Paint Monsters |
| GoNoodle | Crazy Gears | Paint Sparkle |
| Hazel | Cross Fingers | Palace Pet |
| H-T Dash | Cuocake Maker | Paper and Pen |
| Jurassic World Lego | Day Care | Paw Patrol Rescue |
| Just Dance | Dentist | Paw Patrol Take Flight |
| Kirby | Dino Hospital | PBS Parent Play and Learn |
| LEGO Batman | Dinosaur Memory Match | pbskids.com |
| LEGO Creator Island | Discovery Lite | Peppa Pig Shopping |
| LEGO Game | Disney Emoji Blitz | Perler Beard |
| LEGO Indiana Jones | Disney Princess Enchanting | Piano Online |
| LEGO Jurassic World | Disney Story Time | Pikman |
| LEGO Land | Doctor Kids | Pony Care |
| LEGO Nexo Knights | Dolphin Show | Pony Dress Up |
| LEGO Ninjago Skybound | Dr. Panda Airport | Potion Pop |
| LEGO Star Wars | Dr. Panda Day Care | Practice Printing |
| Loonie Racers | Dr. Panda Home | Pre-Programmed Photos App |
| Melinda | Dr. Panda Mall | Preschool ABC’s |
| Plants vs. Zombies 2 | Dr. Panda Space | Preschool Phonics |
| Pokemon | Dragon Ville | Princess Color |
| Pokemon Go | Duolingo for Kids | Princess Juliet |
| Rainforest | Easy Bake | Princess Salon App |
| Redball | Egg Incorporator | Prodigy |
| Sago Forest Flyer | Elmo’s ABC | Puppy Dog Jig Saw |
| Sea Stars | Endless Alphabet | Puzzle Games |
| Sega Racers | Fit Kids Brain Games | Raz Kids Reading |
| Shark Evolution | Follow the Dot | Road Blocks |
| Slime Rancher | FreeFlow | Sago Mini Babies: Doodlecast |
| Spirit Monster | Frozen FreeFall | Sago Mini Babies: Dress Up |
| Strawberry Shortcake Rush | furReal Friends Get Up & GOGO, My Walking Pup | Sesame Street |
| Subway Surfers | furReal Friends Starlily, My Magical Unicorn | Shop Creator |
| Super Mario | Georgia Atkinson | Shopkins |
| Super Mario Galaxy | Happy Teeth | Spite and Mallace |
| Super Mario Kart | Hayday | Splash Math |
| Super Mario Maker | Hello Kitty | Star Wars Puzzle Droids |
| Super Mario World | Hello Kitty Lunch Box | Starfall |
| Tangled | Hooked on Phonics | Stella and Sam |
| Temple Run | Human Body | Story Central |
| The Elements | Inside Out Bubbles | Strawberry Shortcake Bakery |
| Tinker Bell Adventure | Intellijoy Kids ABC | Super Why |
| Titanfall 2 | Jake the NeverLand Pirate | Tailor Kids |
| Tom’s Jet Ski | Joy Preschool | Teach Me |
| Toy Story | Kid’s Handwriting | Tic Tok Clock |
| Transformer Rescue Bots: Disaster Dash | Kids A-Z | Tiny Piano |
| Transformers Rescue Bots: Hero Adventures | Kids Camera | Toca Boca |
| Turbo Dismount | Kids Colouring | Toca Build |
| Up Hill Rush | Kids Puzzles | Toca Hair Salon |
| Vacation | Kids Writing ABC | Toca Lab |
| War Robots | Leap Pad | Toca Life City |
| Yoda Chronicles | Leap Pad 2 | Toca Nature |
| Yoshi’s Wooly World | Leapster | Toca Vet |
| Zelda | Letter Factory | Tracing Letters |
|  | Letter Quiz | UniNumbers |
|  | Letter School | Uno Moos |
|  | Letter Sounds | Veggietales Look and Find |
|  | Letter Train | Wedding Planner |
|  | Letters and Numbers | Yateland |
|  | Littlest Pet Shop Your World | Yo Gabba Gabba |
| **Note:** Fast-reaction games were those that included challenges such as hand-eye coordination and time pressure to make a response. Slow-reaction games were those that involved an educational component such as learning math, the alphabet, or social skills. Games were classified by two independent raters using <http://igdb.com> and other online sources including videos about the games on youtube.com. | | |
