# Supplementary material for: Videogame exposure positively associates with selective attention in a cross-sectional sample of young children
Source: PLoS One. 2021 Sep 27;16(9):e0257877. doi: 10.1371/journal.pone.0257877 (PMC8476027; doi:10.1371/journal.pone.0257877)
Supplement: Attachment — Submitted filename: Response to Reviewers 1.docx [file pone.0257877.s003.docx]

September 25^th^, 2020

Dr. Martinez-Molina

Editor, PLOS ONE

RE: new submission of PONE-D-20-24636

Dear Dr. Martinez Molina,

Thank you for taking the time to consider our work, entitled ‘*Screen-games and their association with cognition and behaviour in young children*’, and provide some constructive feedback. We have attempted to address your feedback in this updated manuscript which is a new submission as a Research Article to PLoS One. The previous submission number was PONE-D-20-24636.

We have made the following changes following your comments on the previous submission:

1) I recommend that you further specify the objectives and hypotheses of your study. For example, differences between sex are included when nothing was stated in this regard in the introduction to the manuscript.... and descriptives about types of video game platform are included, without substantive theoretical relationship.

We thank the editor for pointing out that our examination of sex differences was not well justified in the Introduction. We have now added a paragraph outlining our rationale for examining sex, namely that studies in older children suggest boys spend more time playing screen games and that boys also show higher rates of ADHD and ASD, suggesting that sex is an important variable to consider when assessing associations between screen games and behavior.

We have now removed the description of game platform, as we agree we have insufficient information to draw conclusions about the context based on this information.

2) I particularly miss a bit more order in the results section and the calculation of the effect sizes in the ANOVAs.

We have now added several sub-sections in the Results and divided up tables to improve clarity. We have a new section specifically addressing sex differences in outcome measures. However, we find the effect size comment is confusing. We do include partial eta squared effect sizes when we report ANOVA results throughout the text.

We thank you for taking the time to consider our paper, which examines cognitive and behavioral associations with screen-game use in early childhood. Screen-games are part of daily life for many young children, despite mixed evidence of the risks or benefits, particularly regarding early use. In older children and adults, screen-games have been associated with behavioural challenges such as attention deficit hyperactivity disorder (ADHD), as well as cognitive benefits, such as better selective attention and visuomotor control in young adults who play action video games. However, most studies of screen media activities in young children have considered screen-media under a broad umbrella, not distinguishing between more passive or active forms, therefore potentially missing important nuances in the data.

Here, we sought to describe the types of screen-games used by young children, including the specific games children play, and examine associations with children’s cognition and behaviour with a careful consideration of demographic variables including age, sex and maternal education. We collected cross-sectional data from 155 children aged 4-7 years (77 male). Parents reported on average daily weekday screen-game use, including devices used and specific games played. We used multi-informant methods to collect parent-rated ADHD and Autism Spectrum Disorder (ASD) symptoms, and direct measures of cognition from children including IQ, selective, sustained and executive attention and visuomotor integration. We report the games that children play and whether they place high demand on attention. We found that time spent on screen-games increased across this age range and significantly associated with hyperactivity, but not other cognitive or behavioral measures.

A novel contribution of our work is examine screen-games specifically, including the type of games children spend time playing. We believe this research makes an important contribution to this highly topical area and will be of interest to the readership of PLoS One, which we note has published several important studies in this area (Tamana et al. 2019, Tong et al. 2016).

These findings have not been previously published. The data used in this study were collected as part of a neuroimaging study; findings on functional connectivity and white matter development in this sample have been published (Rohr et al. 2017, 2018, 2019; Dimond et al. 2020).

Sincerely,

**Signe Bray, PhD**

Associate Professor, Department of Radiology

Cumming School of Medicine

University of Calgary
