# Supplementary material for: Videogame exposure positively associates with selective attention in a cross-sectional sample of young children
Source: PLoS One. 2021 Sep 27;16(9):e0257877. doi: 10.1371/journal.pone.0257877 (PMC8476027; doi:10.1371/journal.pone.0257877)
Supplement: Attachment — Submitted filename: Reviewer Response.docx [file pone.0257877.s004.docx]

**Response to Reviewers**

We thank the reviewers for their insightful comments on our manuscript. In response to the feedback, we have made major revisions to our manuscript, including:

- Title change to more accurately reflect the study findings
- Revisions to the Introduction to further emphasize research on similar topics and study rationale
- Clarification of methodology
- Focusing the paper by removing of visuomotor and ASD behaviours from the analyses
- Addition of strengths and limitations to the Discussion

Further, while revising the manuscript, an error in our raw data came to light and therefore some of the associations reported here have changed slightly. The biggest changes are that selective attentions scores are positively correlated with videogame time in this updated manuscript and associations with hyperactivity are non-significant when controlling for maternal education.

Below we have addressed each comment point-by-point. Reviewer questions are **bolded**, text from the manuscript is *“italicized and in quotations”*, with new additions/revisions to the manuscript in blue (including new references in the Reference section) and the corresponding lines highlighted in yellow.

Together, we feel our manuscript has been improved with these revisions and changes, and we are grateful for the reviewer’s time and contribution to this effort, and for PLOS One for receiving our revised manuscript.

**Reviewer #1**

**The authors examine the linkage between young children's time spent playing digital games, diverse indices of their attentional abilities including sustained and selective attention, and ADHD and ASD symptoms.**

1. **The reasons for studying these linkages is not well-justified. In fact, the authors' claims about the relative dearth of information about young children's digital game exposure as linked to cognitive skills reflect limited consultation of developmental research that would show otherwise. Specifically, the authors are urged to consult the work emanating from the labs of, for example, Heather Kirkorian and Ellen Wartella. Consideration of the characteristics of the games and apps that young children use and the cognitive and academic skills that may be promoted through their play also is available in research done by those within the developmental psychology and educational technology communities.**

We agree that the Introduction and justification for the study could benefit from a more in-depth description of the prior literature. We have now substantially revised the Introduction to focus more directly on the main novel contribution of our work, which is direct assessment of attention skills in young children, and also referenced the important work by Wartella and colleagues [14, 19] and other development psychology research on related topics (i.e., Dale, Bavelier & Green (2020) [10]; McNeill et al., (2019) [18]; Jusiene et al. (2020) [21]). Please see the Introduction section for all of the changes made (Pages 3 – 5, Lines 26 -81).

Further, work by Kirkorian and colleagues [46] was added to the Discussion (Lines 434 – 435)

2. **Reasons for linking game play to ASD symptoms is not well grounded in the literature aside from these symptoms being shown more frequently among males than girls; the latter of whom are presumed to spend more time playing digital games than girls. This presumption alone, particularly among young children, warrants greater substantiation than the authors provide.**

We agree that the rationale for examining ASD symptoms was under-developed and have decided to remove measures of ASD symptoms and behaviours from the manuscript.

3. **The methods section was far too limited in the information provided. For example, which parent completed the surveys? Did parents have full knowledge of children's game activities outside the home, given that many preschools may not include digital game play as part of the academic curriculum. Similarly, were games included if they were presented as part of a website that included activities for children?**

We thank the reviewer for noting details that should be added to the Methods section. These questions helped us to improve the clarity of this section.

- **Which parent completed the surveys?**

We have added this to the Procedure of the Methods section: “*During these sessions, parents completed questionnaires (10% were fathers) and children participated in cognitive assessments.”* (Lines 108 –110)

- **Did parents have full knowledge of children's game activities outside the home, given that many preschools may not include digital game play as part of the academic curriculum?**

Great question, *“Parents completed a questionnaire [17] that asked them to estimate their child’s typical weekday time spent playing videogames at home over the past two weeks*.” (Lines 122-123)

- **Similarly, were games included if they were presented as part of a website that included activities for children?**

Parents provided information about devices used to play videogames and names of the videogames played. Based on Google searches of the games that were named by the parents, some were internet games. This has now been added to the manuscript for clarification: *“Videogames included games that were played on the internet, television, or handheld device.”(*Line 123 - 124)

4. **Distinctions between what qualified as action v. non-action game were fuzzy. What exactly would qualify as a non-action game? In looking at Table 2, it seems as if those games that were more educational in nature were more inclined to be labeled as non-action games. What was the inter-rater reliability for making distinctions between the two types of games as none is reported.**

We agree that the distinction between action and non-action games was not clear therefore we now classify the games as attention-demanding and non-attention-demanding games. We believe this classification better captures the way in which we rated the games. For example, as the reviewer mentions the non-attention-demanding games are typically educational in nature thus require less demand of attention.

*“The cognitive psychology literature typically distinguishes ‘action’ from ‘non-action games with action games being games that involve rapid pacing, switching attention between vigilance across the visual field to monitor for potential threats, and focusing in to accomplish a specific task and often include the control of an avatar (e.g. first-person shooter; [10]). However, these types of games are not commonly used by young children therefore we decided to categorize the games as ‘attention-demanding’ and ‘non-attention-demanding’ games. The former involved games with challenges such as fast reaction time, hand-eye coordination, and time pressure to make a response while the latter, involved games with an educational component such as learning math, the alphabet, or social skills.”* (Lines 139 – 143).

Inter-rater reliability was reported: *“There was high inter-rater reliability between videogame classifications with 97% agreement but, in the case of a disagreement, a third rater acted as a tiebreaker.”* (Lines 145 – 147)

5. **What was the reason for including particular subtests of the WPPSI-IV?**

Great question. We added an explanation to the methods section: *“These sub-tests were chosen as representative measures for each of the five WPPSI-IV sub-domains (visual spatial, fluid reasoning, working memory, processing speed, verbal comprehension).”* (Lines 160 – 162)

6. **What exactly did the attention assessments as modeled on ECAB entail? The authors should be willing to provide at least a brief description. How many trials in the selective attention task? What was the basis for the sustained attention task used in the study and who developed it?**

Thank you for the questions, we have elaborated on the ECAB in the Methods section. Specifically, we have clarified how all four of the cognitive attention measures were derived from the ECAB. The ECAB measurement is broadly discussed followed by detailed explanations of the four sub-tests that were used in the study (Lines 166 – 211).

7. **What were the criteria that needed to be met for successful completion of forms in the VMI task?**

We decided to streamline our paper to focus on attention and VMI was removed from the analysis. We also note that because this measure was added after data collection began and therefore was only available for a smaller sample (n = 129), making comparisons to other outcomes reported more complicated.

8. **For the behavioral assessments, were the parents the reporters or were independent assessments made as the authors make mention on page 4 of using a multi-informant approach. In fact, the authors later note in their discussion section that a strength of the study was this approach. Thus, specific mention of these informants is warranted.**

We have decided to use the term multi-method instead of multi-informant to clarify that different types of methods were used to collect the data for this study; parent-reports of ADHD behaviour traits and videogame data in addition to direct assessments of children’s attention and general cognition. We have made this more explicit within the Methods section as well as in the Introduction (e.g. Introduction section: *“As a step towards understanding the potential impact of videogames on attention and cognition in early childhood, we describe a cross-sectional study examining associations between parent-reported videogame use and assessments of children’s attention skills.”* (Lines 68 – 70).

9. **The results section was somewhat baffling to me as I failed to understand why participants with missing or outlier data were still included in the analyses. How were the missing data points handled within the data set? Similarly, why were those participants who exceeded clinical cut-offs on the AQC still included in the study?**

We have now clarified that participants with missing data on a particular assessment were not included in the analyses that included that measure as a predictor or outcome.

For example, if a participant did not have a selective attention score, they were not included in analyses when selective attention was being assessed. Additionally, the one selective attention outlier was removed from the model of videogame play and selective attention scores in order to test whether the effect remained significant (Line 285-287).

We have now added a clarification to the Participants section: *“Data was analyzed for 154 children (77 female) who had complete videogame data; however, not all 154 participants had complete data on maternal education (n = 2), cognitive outcomes (selective attention, n = 4; visual sustain attention, n = 8; auditory sustained attention, n = 5; executive attention, n = 7; IQ, n = 2), and behavioural traits (SNAPI, n = 2; SNAPH, n = 3; SNAPC, n = 3). Participants were excluded from any analyses for which they had missing data.”*(Lines 98 – 103)

Analyses involving the AQC have now been removed from the manuscript.

10. **Overall, I am not sure what story is told by the minimal significant findings or why the study was done. The distinction between action and non-action games seems particularly blunt and the multi-informant approach is not at all obvious.**

As the reviewer suggested and as mentioned above, we added rationale to the Introduction including emphasis on literature of videogames in older children as well as videogames in younger children (see response to #1) to highlight the rationale for conducting this study. We have also clarified the distinction between game characteristics (see response to #4) and clarified what we meant by multi-informant approach (see response to #8).

**Reviewer #2**

**This paper investigates the association between video-games and cognitive measurements and hyperactivity and autistic traits measured by questionnaires filled by parents in preschool children. This is a very well-written and sounded paper that I recommend for publication with minor revisions that I detail here below:**

1. **The title is not very accurate: there is no association between screen-games and cognition and the association with behavior is in fact an association with parents' perception of their children's behavior. I would suggest to put the result: video-games were not associated with cognitive abilities but with behavior reported by parents.**

The reviewer raises an important consideration therefore we decided to change the title of the manuscript to: *Videogame play associates with selective attention skills and hyperactivity in early childhood*

Additionally, we took the reviewers suggestion to clarify the results of the study (i.e., Conclusion section: *“We found a significant positive association between time spent playing recreational videogames and selective attention as well as a significant positive association between time spent playing recreational videogames and parent-rated hyperactivity behaviours. Importantly, our cross-sectional findings cannot be used to infer causality or directionality of these associations and future longitudinal or interventional studies are needed to determine whether playing videogames in early childhood influences cognitive or behavioral development.”* (Lines 442 - 448).

2. **Define what screen-media activities is and what is screen-games. Why the authors do not use the term video-games?**

We appreciate the suggestion and we decided to use videogames, instead of screen-games, throughout the manuscript.

Additionally, examples of screen-media activities were provided: *“There is growing concern about young children’s exposure to screen-media activities (SMAs) [1] such as television or videogames,…”* (Lines 27 – 28).

3. **The introduction can be better structured. Here is my suggestion: begin with definitions, then prevalence and then the effects of games on cognition. Then you can develop in a separate section the effect on hyperactivity and autistic traits. This would help formalize better hypotheses about the association between hyperactivity and autistic symptoms. In fact, I did not fully understand the association between video-games and autistic symptoms.**

As mentioned in the response to Reviewer 1 (response #1), the Introduction was thoroughly revised to more clearly justify the study rationale in light of existing literature. Further, as noted above, analyses of ASD traits have been removed from the manuscript.

4. **In your hypothesis "We hypothesized that time spent playing screen-games would be positively associated with ADHD and ASD symptoms and that playing attentionally demanding games would be associated with better selective attention and visuomotor integration", how can you reconcile a positive effect on cognition and a negative effect on behavior?**

We agree that these hypotheses may sound counterintuitive; however, directly assessed cognitive skills and parent-reported behaviour traits do not always correlate. In the ADHD literature, there is variation in the extent and nature of cognitive challenges reported. In context of our study hypotheses, we considered that there could be a possibility that a child who scores high on parent-rated hyperactive traits and frequent videogame usage, could also demonstrate high selective attention abilities through extensive practice from frequent videogaming.

We have now added a sentence to justify the reason for our hypothesis as well as cited other resources that have found similar results: *“It may be counterintuitive to hypothesize different direction of effects when considering cognitive or behavioural measures, but these hypotheses are supported by previous literature [8, 24]. Further, children in our sample did not have a diagnosis of ADHD and the literature on cognitive differences in ADHD is mixed, with few studies examining cognitive differences specific to inattentive or hyperactive ADHD traits [25].”* (Lines 76 – 81)

5. **Hyperactivity and autistic traits were studied through questionnaires by parents and these were the only significant correlations with screen-games. This was not discussed in the discussion. How reliable are parents' answers? This study shows a correlation between games and parents' perception of the level of activity of their children and some of their personality traits. This highlight should have implications on the title, the abstract and the discussion.**

Many great points are made here. We have made sure to be more explicit in mentioning that the behaviour outcomes are based on parent reports in the Title, Abstract, and Discussion (e.g., Abstract section: *“We found that videogame time was significantly positively associated with directly assessed selective attention and parent-rated hyperactivity scores, but not parent-rated inattention or other directly assessed attention skills.”* (Lines 11 – 13).

Also, we have added the parent-reports as a limitation to the study in the Discussion of the manuscript: *“Finally, parent- reports likely underestimate actual use of videogames and mobile devices in young children [45]. Future work should incorporate objective measurements for more accurate videogame usage including time-use diaries and passive sensing [46] in addition to, teacher reports of children’s ADHD behaviours to corroborate the parent reports [47].”* (Lines 432– 436)

6. **The table with demographics is good and could contain the percentages so the authors do not repeat the figures in the paragraph below.**

Following this suggestion we eliminated the percentages in the text. Now all percentages can be found in Table 1 (Line 250)

7. **The table with the distinction between action games and non action games is not very interesting. You may give an example.**

The Table with the list of action and non-action games was removed. As per the response to Reviewer 1 (response #4), the games are now classified as attention-demanding and non-attention-demanding. An example of an attention-demanding game and a non-attention-demanding game were provided in the text instead: *“There were 226 different videogames reported that were classified as attention-demanding (n = 72) or non-attention-demanding (n = 154). For example, Subway Surfers [31] was considered an attention-demanding game while My Little Pony: Friendship Gardens [32] was considered a non-attention-demanding game.”* (Lines 262 – 265)

8. **There is a sentence in the discussion saying that this study is correlational and other studies need to study the causal effects. I agree and in my view this is not sufficiently developed in the discussion especially when showing the positive correlations between games and hyperactivity and autistic traits. My concern is that this may mislead some readers or can be misinterpreted by journalists and parents.**

We understand the concern, and have made sure to be clear when explaining that this study is correlational not causal whenever necessary through out the manuscript. For example, we acknowledge the correlational nature of this work in the new Title, Introduction, Results, and Discussion sections to more accurately reflect the results of the study.
