# Supplementary material for: Videogame exposure positively associates with selective attention in a cross-sectional sample of young children
Source: PLoS One. 2021 Sep 27;16(9):e0257877. doi: 10.1371/journal.pone.0257877 (PMC8476027; doi:10.1371/journal.pone.0257877)
Supplement: Attachment — Submitted filename: Response to Reviewers.docx [file pone.0257877.s005.docx]

**Response to Reviewers**

We thank the reviewers again for their valuable feedback on our manuscript, which we have integrated into this revised submission. We have incorporated most of the suggestions made by Reviewer #1. The reviewers questions are **bolded**, text from the manuscript are *“italicized and in quotations”*, with new additions/revisions to the manuscript in *blue* and the corresponding lines are highlighted in yellow. Please see below for a point-by-point response.

Altogether, we feel this new revision of the manuscript encompasses the key contributions of our study and is therefore more focused and concise. Again, we would like express our gratitude towards the reviewers for their positive contribution to this manuscript.

**Reviewer #1:**

1. **I remain unconvinced that the changes here help to better substantiate the need for the study. For example, I fail to understand how the study shared here provides nuanced information to address mixed findings in the data concerning the benefits or liabilities of SMAs which sometimes seem to refer to videogames and sometimes to a larger category of screen devices.**

In light of this and comment #3, we have substantially revised the manuscript beginning with the Introduction, to focus on the main contributions of our study, which is associating recreational videogame exposure with attention skills in an early childhood sample. We acknowledge the confusion and inconsistency in using both screen-media activities (SMA) and videogames therefore, we now mention SMA once in the Introduction to illustrate that videogaming is a type of SMA: “*There is growing concern about young children’s exposure to screen-media activities (SMA)* [1,2], *such as television or videogames, as excessive exposure may displace time from other developmentally important activities and could impact brain, behaviour, and cognitive development* [3–6].” (Lines 23 – 26) and once in the Conclusion to suggest that our findings can support videogaming guidelines for young children thus, help guide children SMA guidelines as a whole: *“Videogaming is increasingly common in young children and to support recommendations on SMA use for families it is important to understand whether videogaming associates with children’s attention skills and whether associations are positive or negative.”* (Lines 357 - 359).

2. **I am no clearer on what constitutes attention-demanding v. non-attention demanding which is likely the eyes of the player rather than the investigators. Without far greater substantiation, I do not buy into the assumption that action games are attentionally demanding and educational games not, especially for younger children.**

Our goal was to draw parallels to literature in older children and adults suggesting that there is a specific benefit of action games in terms of attention skills. However, we acknowledge that we used internet descriptions of games for classification and did not do a detailed content analysis of each game, nor have our definitions of different types of games been validated. However, we felt that the detail we collected in terms of games children play was valuable and worth exploring. We have now therefore moved the analysis based on game content to sections that are explicitly labeled ‘exploratory’ on page 10 in the Methods section (Lines 174 – 193) and pages 13 and 14 in the Results section (Lines 254 – 271).

Moreover, to better reflect the videogames that were played by the children the games are now referred to as ‘fast-reaction games’ and ‘slow-reaction games’ rather than ‘attention demanding’ and ‘non-attention demanding’ (Lines 182 – 185).

3. **I do not understand why the examination of ADHD "traits" and why the display of those traits at a non-clinical level is necessarily bad. Again, why only parent informants here warrants explanation.**

This part of the study was a replication of prior work with less informative data. Given the limited information collected here on parenting and family environment, we have now removed analyses related to attention-deficit hyperactivity disorder (ADHD) traits from the manuscript to focus our study on attention skills more specifically.

4. **I remain unclear about what is meant by attention skills, which is not a monolithic construct or why selective attention in particular is targeted within the hypothesis. The methods section does make it clearer that different forms of attention are examined. However, reasons for their investigation remain unspecified in the study rationale.**

In terms of study rationale, we have now clarified through the Introduction that because children spend a lot of time playing videogames, which place demands on attention skills, there is an opportunity for experience-dependent plasticity and the potential for games to associate with attention skills as a result. Specifically, we hypothesize that selective attention, the ability to focus and ignore distractions, may be enhanced in children who spend more time playing videogames.

We now more clearly articulate in the Introduction that this study used a tripartite model of attention and the rationale for looking at attention in relation to videogames: *“Attention is a multi-faceted construct that has been conceptualized in a tripartite model including sustained, selective, and executive components* [25-27]*. All three components are maturing in young children* [25], *perhaps conferring an opportunity for experience-dependent developmental plasticity with videogame use. Spending time playing videogames that engage attention may therefore associate with greater skills in these areas. Further, with a growing interest in the use of ‘serious-games’ (e.g., educational videogames used to enhance working memory)* *for cognitive therapeutic purposes* [28]*, it is valuable to determine whether videogames that place demands on vigilance and fast-reaction* *played recreationally are associated with cognitive benefits in young children.”* (Lines 41 – 49).

We now more clearly articulate our reasoning for this hypothesis in the Introduction: *“Based on prior literature in young adults* [23,29], *we hypothesized that time spent playing videogames would be associated with better selective attention.”* (Lines 53 – 55).

Additionally, in the Methods section we specified that the Early Childhood Attention Battery (ECAB) was used to assess three components of attention in line with the tripartite model. *“Children completed eight sub-tests of the ECAB, four of which were included in this study to assess the three components of attention: selective attention, sustained attention (visual sustained attention and auditory sustained attention), and executive attention.”* (Lines 115 – 118).

5. **I was further confused as to why maternal education was examined and how game play was assessed. For example, if children are enrolled in day care or some preschool program, might it be the case that children play digital games in those settings? For that matter, might they play games on apps with their friends or a sibling? I ask as I find it unusual that so many children would be characterized as "no-gamers." Regardless, the distinction between types of games children play is no clearer to me than in the earlier version of the study.**

As noted in the manuscript (Lines 85- 89) maternal education was used as proxy for socioeconomic status: *“**For analyses presented here, maternal education (highest degree completed) was used as an indicator of family socioeconomic status (SES) and was grouped into five categories: high school diploma, technical/trade school degree, college diploma, bachelor’s degree, or graduate/professional school.”*

We are now clearer that we assess only recreational game play in the home. For example, in the Introduction: *“The goal of the present study is to assess whether there are associations between videogame exposure and attention skills in young children. Specifically, this study examined associations between parent-reported weekday recreational videogame use and direct assessments of children’s selective, sustained, and executive attention skills.”* (Lines 50 – 53). As well as in the Conclusion: *“Finally, parent- reports likely underestimate actual use of videogames and mobile devices in young children [35] as well as, only recreational games were included in this study therefore games played in school, at a friend’s house, or elsewhere were not taken into account.”* (Lines 349 – 352).

As noted above, we have removed the attention-demanding game distinction from the main analyses to an exploratory analysis as well as renamed the games to better characterize the games played by the children (please see response to comment #2).

6. **I can appreciate the authors' goals to analyze data that they have about children's game play and individual differences but a better reason for doing so needs to be apparent as do far clearer measures and characterizations of the games played for readers to identify the story being shared by the findings.**

We have taken this feedback to heart and as noted above have further streamlined the paper to focus on the key contributions: weekday time spent playing recreational videogames and selective attention skills in typically developing young children. As mentioned above in comment #5, we have attempted to be more clear in introducing the three measures of attention that are being assessed according to the tripartite model of attention. Additionally, as mentioned in the manuscript, a validated battery of attention was used to measure the three types of attention which is more than what other studies have done. Furthermore, as there are no standardized and validated parent reports or other measures for videogame use in young children we would have used them but as this is a rapidly evolving field validated measures have yet to be established. For these reasons, we made the analyses regarding videogame content and their characterization exploratory given the lack of prior literature guiding the distinction between types of games. Overall, our approach mirrors and extends on previously published research and provides insights into a very timely topic, at a crucial time in children’s cognitive development.

**Reviewer #2:**

**The authors have now answered all of my questions and the article has been revised accordingly. I believe that the paper can be published now.**
